# A comparative study of the effectivity of MSC-based, NP-based and combined therapies in an experimental model of NaIO_3_-induced retinal degeneration

**DOI:** 10.1038/s41598-025-07727-7

**Published:** 2025-07-01

**Authors:** Katerina Palacka, Barbora Hermankova, Tereza Cervena, Pavel Rossner, Vladimir Holan, Eliska Javorkova

**Affiliations:** 1 Department of Toxicology and Molecular Epidemiology, Institute of Experimental Medicine of the Czech Academy of Sciences, 14220 Prague 4, Czech Republic; 2 Department of Cell Biology, Faculty of Science, Charles University, 12843 Prague 2, Czech Republic

**Keywords:** Retina, Mesenchymal stem cells, Retinal microglia, Silver nanoparticles, Retinal diseases, Tissue engineering and regenerative medicine, Neuroimmunology, Mesenchymal stem cells

## Abstract

Mesenchymal stem cells (MSCs) represent the promising options for retinal therapy and combined therapy with nanoparticles (NPs) could currently provide increased immunoregulatory and neuroprotective effects. Therefore, we tested the effect of silver (Ag)NPs on the properties of MSCs in an experimental model of chronic retinal degeneration. The results showed that simultaneous administration had no effect on the survival of MSCs, but a less effective local regulation of *Iba-1* expression compared to MSC- or AgNP-only treated groups was observed. In addition, MSCs applied alone or in combination with AgNPs and sorted from the degenerated retina had increased expression of genes for retinal markers (rhodopsin, S-antigen, recoverin), and for TGF-β and IGF-1. These effects were confirmed also on protein level by increased production of IGF-1 and proportion of rhodopsin^+^ MSCs. Nevertheless, the increased expression of the gene for GDNF was observed only in the MSCs combined with AgNPs. Regarding the immune response, the application of MSCs with AgNPs triggered increased expression of the *IL-6* gene in the CD45 cells separated from the retina. In conclusion, applications of MSCs or AgNPs, as a single therapy, were able to modulate the inflammation. However, the combined applications decreased the immunomodulatory effects of MSCs or AgNPs.

## Introduction

Mesenchymal stem cell (MSC)-based therapy represents a potential option for the treatment of several types of currently uncurable diseases. MSCs produce numerous types of immunomodulatory and growth factors, which contribute to the regulation of the inflammatory response during disease or injury and improve tissue healing^1–3^. Moreover, MSCs possess the ability to differentiate into various cell types^4,5^ and support the replacement of degenerated and non-functional cells. It has been shown that changes in the expression and production of immunomodulatory and growth factors by MSCs are dependent on their environment and stimulation^2,3,6–8^. For example, MSCs react to an inflammatory environment by changes in the expression of genes for nerve growth factor (NGF), glial cell line-derived neurotrophic factor (GDNF), cyclooxygenase-2 (Cox-2), indoleamie-2,3-dioxygenase (IDO), TNF-α stimulated gene 6 protein (TSG-6), programmed death-ligand 1 (PD-L1), insulin-like growth factor 1 (IGF-1), fibroblast growth factor (FGF), epithelial growth factor (EGF), hepatocyte growth factor (HGF) or transforming growth factor-β (TGF-β)^2,3,7^.

Recently, nanoparticles (NPs) have become widely used in medicine due to their unique properties^9^. NPs have been suggested as antibacterial agents, diagnostic markers and drug delivery vehicles. Several studies have also shown that NPs interact with the immune system and downregulate the function of various immune cells^10–12^. In diseases where an aberrant inflammatory reaction plays a crucial role, inhibition of the immune reaction by NPs could provide a novel therapeutic option.

Retinal degenerative diseases remain one of the main causes of vision loss in adult patients. Current treatment protocols are very limited and only slow down the progression of the degeneration^13,14^. The most common retinal degenerative disorder is represented by age-related macular degeneration (AMD), affecting several millions of elderly patients^15–17^. Other types of these ophthalmological diseases with similar pathological changes are for example diabetic retinopathy (DR), the complication associated with the development of diabetes mellitus, or retinitis pigmentosa (RP), the group of inherited retinal diseases which are associated with retinal dystrophy. A reduction or loss of function of specialized retinal cells, mainly retinal pigmented epithelial (RPE) cells and photoreceptors, are the main markers of retinal damage^18–20^. In recent years, several studies have highlighted the importance of an upregulated immune reaction in the retina as one of the major factors contributing to the progression of disease^21–25^. Activated microglia and macrophages accumulate in the degenerated retina and play an important role in the development of inflammatory conditions in the eye. Microglia and macrophages in a proinflammatory state express higher levels of Iba-1 and CD45 markers and produce several factors, such as cytokines from the interleukin-1 (IL-1) family, galectin-3, nitric oxide and tumor necrosis factor-α (TNF-α) and contribute to the amplification of the immune reaction in the retina^3,26–30^.

The application of MSCs as a potential treatment of a variety of types of retinal diseases has been tested in several experimental studies and clinical trials^1^. The studies and trials were focused on the treatment of ophthalmological condition in general, including AMD, DR, RP, glaucoma, optic nerve diseases etc^1,31,32^. Even though the experimental results are promising, there are still several limitations, for example activation of immune response as a result of invasive application or rapid phagocytosis of transplanted MSCs by retinal microglia or macrophages. Moreover, the paracrine effects and production of specific immunoregulatory and regenerative factors by MSCs is dependent on the stimulation^2–4^. Thus, the combination of stem cell-based treatment with other types of therapy could potentially provide better outcomes. The first experimental studies using nanomaterials in ophthalmology were published in the last decade^33–37^. Although some in vitro tests reported possible toxicity of NPs for retinal cell lines, several in vivo studies showed a positive effect of the NPs in the treatment of retinal diseases^33–38^. We have also observed that application of silver (Ag) NPs provides modulation of the immune reaction in a mouse experimental model of retinal inflammation^37^. Although MSCs possess immunomodulatory properties in vitro and in the inflammatory retinal environment^3,6^, their local application into the eye with degenerated retina could increase the infiltration of immune cells, as a reaction to the injection. Moreover, the applied cells could be phagocyted by the infiltrated activated macrophages. The combination of MSC-based therapy as a regenerative and neuroprotective agent and AgNPs as anti-inflammatory agents could provide better treatment protocol for retinal diseases.

To date, there is a limited number of studies combining MSCs and NPs as a therapeutic option. The main limitation could be the negative effect of NPs on the properties of MSCs^39,40^. Therefore, in this study, we have focused on the effects of AgNPs on the immunomodulatory and regenerative properties of MSC-based therapy for retinal degeneration. Our results showed that the application of MSCs or AgNPs regulates inflammation in the degenerated retina, however the combined application has a limited effect when compared to a single therapy. In addition, we tested the changes in the expression of genes for immunomodulatory and neuroprotective factors and for retinal markers in MSCs and MSCs applied with AgNPs sorted from the degenerated retinas. MSCs sorted from the degenerated retinas increased the expression of genes for TNF-β and IGF-1 and retinal cell markers. There were no significant differences in the gene expression of retinal cell markers between MSCs applied alone and MSCs applied with AgNPs, but the expression of growth factors *NGF* and *GDNF* was higher in the cells injected in the presence of NPs.

## Methods

### Preparation of AgNPs

AgNPs were purchased as an uncoated silver nanopowder (Sigma-Aldrich, St. Louis, MO, USA). The preparation of AgNP stock dispersions have been described in detail elsewhere^41^. AgNPs were sonicated and dispersed to a final concentration of 2.56 mg/ml and diluted in culture medium for the required concentrations used in the experiments. The material characterization of AgNPs is reported in our previous manuscript^40^.

### Animals

Female mice of inbred strain BALB/c (at the age of 8–15 weeks) obtained from company Envigo (Indianapolis, IN, USA), were used in the experiments. The use of animals was approved by the Local Ethical Committee of the Institute of Experimental Medicine of the Czech Academy of Science, Prague (approval code 6130/2022). All experiments were carried out in accordance with relevant guidelines and regulations. All methods were reported in accordance with ARRIVE guidelines. Acclimatization periods were at least 10 days before any procedure in animal housing. Mice were divided randomly, 10 mice per cage and the cage locations were same for each experimental group. Each types of treatments were provided in the same time. During the experimental procedure, mice were observed for any side effects or changes in their conditions.

### Isolation of MSCs

Small cut pieces from inguinal fat pads from mice were digested at 37 °C with a solution of collagenase I (Sigma-Aldrich, cat.# C2674) in Hanks’ balanced salt solution (HBSS) with Ca^2+^ and Mg^2+^. After the 45-min digestion process, the cell suspension was centrifuged twice at 250 g for 8 min in Dulbecco’s modified Eagle medium (DMEM, Sigma-Aldrich, cat.# D6429) containing 10% fetal bovine serum (FBS, Sigma-Aldrich, cat.# F7524), antibiotics (100 U/ml of penicillin, 100 µg/ml of streptomycin) and 10 mM HEPES buffer (referred to as complete DMEM). Single cell suspension was seeded in a 75-cm^2^ tissue culture flask (Techno Plastic Products, TPP, Trasadingen, Switzerland, cat.# 90075) in DMEM. After a 48-h cultivation, nonadherent cells were gently washed out and the remaining adherent cells were cultivated at 37 °C in an atmosphere of 5% CO_2_. The cells were cultivated for another week, harvested in the 3rd passage and used in our experiments.

### Differentiation potential of MSCs

MSCs were cultivated for 2–3 weeks in a complete DMEM supplemented with specific adipogenic reagents (0.1 µM dexamethasone, 0.5 mM 3-isobutyl-1-methylxanthine cat.# I5879-100MG, 0.1 mM indomethacin cat# I7378, and 0.5 µg/ml of insulin cat.# I6634, all Sigma-Aldrich) or osteogenic reagents (0.1 µM dexamethasone, 0.1 mM L-ascorbic acid cat.# A4544 and 10 mM β-glycerolphosphate disodium salt pentahydrate cat# 50020, all Sigma-Aldrich). The differentiation potential of the cells was evaluated by staining with Oil Red O (Sigma-Aldrich, cat.# O0625-25G) or Alizarin Red S (Sigma-Aldrich, cat.# A5533-25G) or by RT-PCR analysis.

### Cultivation of MSCs in the presence of AgNPs

MSCs following 3rd passage were seeded into 24-well plates (TPP) (10 000 cells/well) and cultivated for 7 days in 1 ml of DMEM and in the presence of different concentrations of AgNPs (0–2.5 µg/ml). On day 7, cells were transferred into TRI reagent (Molecular Research Centre, Cincinnati, OH, USA, cat.# TR118) and prepared for detection of gene expression by real time polymerase chain reaction (RT-PCR). For the determination of metabolic activity, water-soluble tetrazolium-1 (WST-1, Roche, Penzberg, Germany, cat.# 11644807001), was added into each well (at a concentration according to the manufacturer’s instructions) and incubated for an additional 2 h. The supernatants were transferred into 96-well plates (TPP, cat.# 92696) and the optical density was measured in Sunrise Remote ELISA reader (Gröding, Austria) at a wavelength of 450 nm.

In another set of experiments, MSCs were cultivated with AgNPs (for 7 days). Forty eight hours prior to the end of the incubation, proinflammatory cytokines TNF-α, IL-1β and IFN-γ (all from PeproTech, Rocky Hill, NJ, USA, cat.# 315–01 A; 211-11B; 315-05) at a final concentration of 10 ng/ml, were added into each well. At the end of the incubation period, the cells were analyzed by WST-1 test, as described previously, or harvested into 500 µl of TRI reagent and prepared for the detection of gene expression by RT-PCR.

### Induction of chronic retinal degeneration

For the induction of retinal cells degeneration, mice were injected intraperitoneally (i.p.) with NaIO_3_ (Sigma-Aldrich, cat.# S4007) dissolved in phosphate-buffered saline (PBS), at a final dose of 25 mg/kg of their body weight. Application was repeated at intervals of 7 days, and each mouse was injected 3 times in total. Mice were able to tolerate the selected dose without changes in behavior or body weight. During the study, we did not observe any side effects of the repeated intraperitoneal applications. On day 21, after the first NaIO_3_ application, the retinas were isolated from the euthanized mice. The eyeballs were enucleated and the cornea, the lens and vitreous were removed. The retina was dissected from the eyeball and used for flow cytometry and RT-PCR analyses or in further experiments. Due to the small amount of retinal tissue, we used separated group of mice for each analysis.

### Cultivation of the retina with MSCs or MSCs and AgNPs

MSCs were seeded in 24-well plates (10 000 cells/well) in 1 ml DMEM or in 1 ml DMEM and AgNPs at a concentration of 0.025 µg/ml (MSCs/AgNPs). On day 5 of incubation, the medium was exchanged by RPMI 1640 medium (Sigma-Aldrich, cat.# R8758) containing 10% FBS, antibiotics (100 U/ml of penicillin, 100 µg/ml of streptomycin) and 10 mM HEPES buffer (referred as a complete RPMI 1640 medium). The retinas were isolated from NaIO_3_-treated mice and cultured with or without MSCs in RPMI 1640 medium in the presence or absence of 0.025 µg/ml AgNPs. A retina isolated from a healthy mouse was cultured in RPMI 1640 medium and used as a control. Retinas were cultivated on 0.22 μm semipermeable membrane (Nunc, Roskilde, Denmark, cat.# 40652) to prevent direct contact of MSCs with retinal tissue. After a 48-h cultivation, retinas were transferred into 500 µl of TRI reagent and analyzed by RT-PCR.

### Intravitreal application of MSCs and AgNPs

BABL/c mice with induced chronic retinal degeneration were anesthetized using an i.p. injection of 0.15 ml xylazine (xylazinum hydrochloridum 2%, Bioveta, Ivanovice, Czech Republic, cat.# 35630) and 0.15 ml ketamine (ketaminum hydrochloridum 5%, Bioveta, cat.#35095). The intravitreal application of MSCs, AgNPs or serum free medium was performed using a Hamilton syringe (5 µl of volume, Hamilton, Reno, NV, USA, cat.# 7634-01) with a 33G sharp needle (Hamilton, cat.# 7803-05).

Forty-eight hours before the last application of NaIO_3_, experimental mice were divided into 4 groups (untreated, MSCs treated, AgNPs treated, MSCs/AgNPs treated) and 1 control group without application of NaIO_3_. The total number of animals used in the study was 137. 30 mice divided into 5 groups were used for the evaluation of cell populations by flow cytometry, 30 mice divided into 5 groups were used for the evaluation of gene expression in the retina and 15 mice divided into 5 groups were used for the microscopy. For the evaluation of persistence of applied cells in the eye (day 2 and 7) or for the sorting of labelled cells (day 7), 29 mice received PKH26-labelled MSCs and 29 mice received PKH26-labelled MSCs with AgNPs and 4 mice were untreated.

All types of treatment were diluted in serum free medium. The intravitreal applications were performed into the right eye of mice. The first group received 0.5 µl of MSC suspension (10 000 cells), the 2nd group received 0.5 µl of solution of 0.025 µg AgNPs, the 3rd group received 0.5 µl of MSC suspension with solution of 0.025 µg of AgNPs (MSCs/AgNPs) and the 4th group received 0.5 µl of serum free medium. For all analysis, the mice were euthanized on day 7 after the intravitreal applications.

For the analysis of MSC persistence in the retina and sorting of labelled MSCs from degenerated retina, the cells were stained prior to application with vital dye PKH26 (Sigma-Aldrich, cat.# PKH26GL-1KT) according to the manufacturer’s instructions and were applied intravitreally into the eye with the degenerated retina.

Prior to the application, the fluorescence intensity of the staining was tested by flow cytometry using an LSRII flow cytometer (BD, Biosciences, Franklin Lakes, NJ, USA) and by fluorescence microscopy. Cover glasses with grown PKH26-labeled MSCs were fixed for 1 h with 4% formaldehyde (Lachema, Brno, Czech Republic) and mounted in Vectashield mounting medium with DAPI (Vector Laboratories, Inc., CA, USA, cat.# H-1200-10). Images were taken using Zeiss Axioskop microscope, software Isis MetaSystems.

Mice were euthanized on day 2 or on day 7 (for analysis of MSC persistence) and on day 7 (for sorting of labelled MSCs) after the intravitreal applications.

### Preparation of a single cell suspension from the retina for flow cytometry analysis

The isolated retina was homogenized and digested at 37 °C in the presence of 1 mg/ml of collagenase I in HBSS. After a 45-min incubation period, digestion was stopped by adding the excess of RPMI 1640 medium, and cell suspension was filtrated and washed by centrifugation (8 min 250 g).

### Flow cytometry

Retinal single cell suspension or MSCs were incubated for 30 min in 4 °C with anti-mouse monoclonal antibodies conjugated with allophycocyanin (APC), phycoerythrin (PE) or fluorescein isothiocyanate (FITC). A list of the used antibodies (all from BioLegend, San Diego, CA, USA) is shown in (Table 1). Death cells were stained 10 min prior to the flow cytometry analysis by adding Hoechst 33,258 fluorescent dye (Invitrogen, Carlsband, CA, USA). For detection of the rhodopsin-positive MSCs, cell suspension from retina was labeled 30 min at 4 °C with Live/Dead Fixable Violet Dead Cell Stain Kit (Life Technologies, Carlsbad, CA, USA) dissolved in PBS (1:200). Cells were fixed for 30 min at 4 °C with 100 µl IC Fixation Buffer (Invitrogen, cat.# 00-822-49) and then labeled for an additional 30 min with anti-rhodopsin monoclonal anti-mouse antibody (Abcam, Cambridge, United Kingdom, cat.# ab183399).

Data was collected by LSRII flow cytometer and analyzed by FlowJo 9 (Tree Star, Ashland, OR, USA) or the retinal samples were sorted by BD Influx (BD Biosciences) and transferred into 500 µl of TRI reagents.

**Table 1:** Antibodies for flow cytometry.

Antibody	Fluorochrome	Clone	Catalogue number	Manufacturer
anti-CD11b	APC	M1/70	101,212	BioLegend
anti-CD14	PE	Sa14-2	123,310	BioLegend
anti-CD19	FITC	6D5	115,506	BioLegend
anti-CD34	PE	HM34	128,609	BioLegend
anti-CD45	FITC	30-F11	103,107	BioLegend
anti-CD73	APC	TY/11.8	127,209	BioLegend
anti-CD79α	APC	F11-172	133,105	BioLegend
anti-CD90	FITC	30-H12	105,305	BioLegend
anti-CD105	PE	MJ7/18	120,408	BioLegend
anti-CX3CR1	APC	SA011F11	149,007	BioLegend
anti-Galectin-3	PE	Gal397	125,405	BioLegend
MHC II	FITC	NIMR-4	11-5322-85	eBioscience
anti-P2RY12	PE	S16007D	148,703	BioLegend
anti-rhodopsin	FITC	4D2	ab183399	Abcam

### Detection of gene expression by RT-PCR

The total RNA was isolated from MSCs and retinas by TRI reagent according to the manufacturer’s description. Reverse transcription was performed with deoxyribonuclease I (Promega, Madison, WI, USA, cat.# M6101) in DNase I buffer (Promega, cat.# M6101) and the first cDNA strand was synthetized with random primers (Promega, cat.# C1181) using M-MLV reverse transcriptase (Promega, cat.# M1705). The total volume of reaction mixture was 25 µl. Quantitative RT-PCR was performed using power SYBR Green PCR Master Mix (Applied Biosystems, Foster City, CA, USA, cat.# 4367659) by StepOne-Plus Real-Time PCR (Applied Biosystems) with parameters including denaturation at 95 °C (3 min) followed by 40 cycles at 95 °C (20 s), annealing at 60 °C (30 s) and elongation at 72 °C (30 s).

Fluorescence data were collected at each cycle after the elongation at 80 °C for 5 s. The collected data was analyzed by StepOne Software version 2.3 (Applied Biosystems). A comparison with *glyceraldehyde 3-phosphate dehydrogenase* (*GAPDH*) gene was used for the calculation of relative expression of the analyzed gene. The primers used for amplification are shown in (Table 2).

**Table 2:** Sequences of oligonucleotides used in RT-PCR.

Gene	Forward primer	Reverse primer	Gene accession #	Length of the amplified fragments
*Adipsin*	CACGGAAGCCATGTAGGG	CTGGGAGCGGCTGTATGT	NM 001291915	90
*bFGF*	AGCCGTCCATCTTCCTTCAT	CTTCTTCCTGCGCATCCATC	NM 008006	159
*Galectin-3*	TGCGTTGGGTTTCACTGTGCC	GGTGCCCTATGACCTGCCCT	NM 010705	82
*GAPDH*	AGAACATCATCCCTGCATCC	ACATTGGGGGTAGGAACAC	NM 001289726	109
*GDNF*	GACATCCCATAACTTCATCTTAGAGTC	TCCAACTGGGGGTCTACG	NM 001301332	77
*Iba-1*	CAGCATTCGCTTCAAGGACATA	ATCAACAAGCAATTCCTCGATGA	NM 001409899	144
*IGF-1*	ACGACATGATGTGTATCTTTATTGC	TCGGCCTCATAGTACCCACT	NM 001111274	92
*IL-1β*	AGCTGGATGCTCTCATCAGG	AGTTGACGGACCCCAAAAG	NM 008361	75
*IL-6*	CCAGGTAGCTATGGTACTCCAGAA	GCTACCAAACTGGATATAATCAGGA	NM 001314054	78
*NGF*	TGGACTGCACGACCACAG	AAATTAGGCTCCCTGGAGGT	NM 013609	68
*Osteocalcin*	CTCGTCACAAGCAGGGTTAAG	AGACTCCGGCGCTACCTT	NM 001032298	93
*Osteopontin*	TGCCAGAATCAGTCACTTTCAC	GGAGGAAACCAGCCAAGG	NM 001204201	159
*PPARγ2*	GGGGGTGATATGTTTGAACTTG	GAAAGACAACGGACAAATCACC	NM 011146	92
*Rcv*	AGGGTCCCCTCGATGAAT	AGATCTGGGCATTCTTTGGA	NM 009038	70
*Rlbp*	TTTGAACCTGGCTGGGAAT	CCCCTCGGATCTCAAGAAG	NM 001173483	61
*Rhodopsin*	TGCCCTCAGGGATGTACC	ACCTGGATCATGGCGTTG	NM 145,383	70
*S-ag*	CACCAGGATCCCCATGAC	AAGCATGAGGACACAAACCTG	NM 009118	78
*TGF-β*	TGGAGCAACATGTGGAACTC	CAGCAGCCGGTTACCAAG	NM 011577	71
*TNF-α*	GCTCCAGTGAATTCGGAAAG	GATTATGGCTCAGGGTCCAA	NM 013693	143

### Enzyme-linked immunosorbent assay (ELISA)

Seven days after intravitreal application of treatment, MSCs were separated from the degenerated retina and were cultured in 96-well plate (8 000 MSCs per well in 200 µl of complete DMEM) for 48 h. Concentrations of IGF-1 in supernatants were quantified using ELISA kits purchased from R&D Systems (Minneapolis, MN, USA, cat.# DY791) according to the manufacturer’s instructions. The concentration was quantified using Sunrise Remote ELISA Reader.

### Fluorescence microscopy of retina

Eyeballs were enucleated and immediately fixed with 4% paraformaldehyde for 1 h and additional overnight cryoprotection in 15% sucrose (Sigma-Aldrich, cat.# S0389-500G). The eyeballs were embedded in Tissue-Tek O.C.T. Compound (Sakura Finetek, cat.# 4583). Frozen sections at the thickness of 10 μm were prepared using Cryostat Leica CM1950, fixed with 4% paraformaldehyde for 10 min and washed with PBS 2 times. For the detection of rhodopsin, samples were blocked with 5% BSA (Sigma-Aldrich, cat.# A7030-10G**)** for 1 h, stained with the anti-rhodopsin antibody (Abcam, cat.# ab183399) in permeabilization buffer (Invitrogen, cat.# 00-8333-56) for 1 h and mounted in Vectashield antifade mounting medium with DAPI and phalloidin (Vector Laboratories, cat.# H-1600-10, H-1200-10). For the detection of CD11b positive cells, samples were blocked with 5% BSA for 1 h, stained with the anti-CD11b antibody in PBS for 1 h and mounted in Vectashield antifade mounting medium with DAPI. Mounted samples were analyzed by laser scanning confocal microscope Olympus FV1000 and analyzed by Olympus Flouroviewer FV10-ASW 4.2.

### Statistical analysis

The results are expressed as the mean + SD. Comparisons between the two groups were analyzed by Student’s t-test and multiple comparison was analyzed by ANOVA. A value of

*p* < 0.05 was considered statistically significant. GraphPad Prism 9 Software (Dotmatics company, United Kingdom) was used for the statistical analysis.

## Results

### Characterization of MSCs

**Fig. 1:**
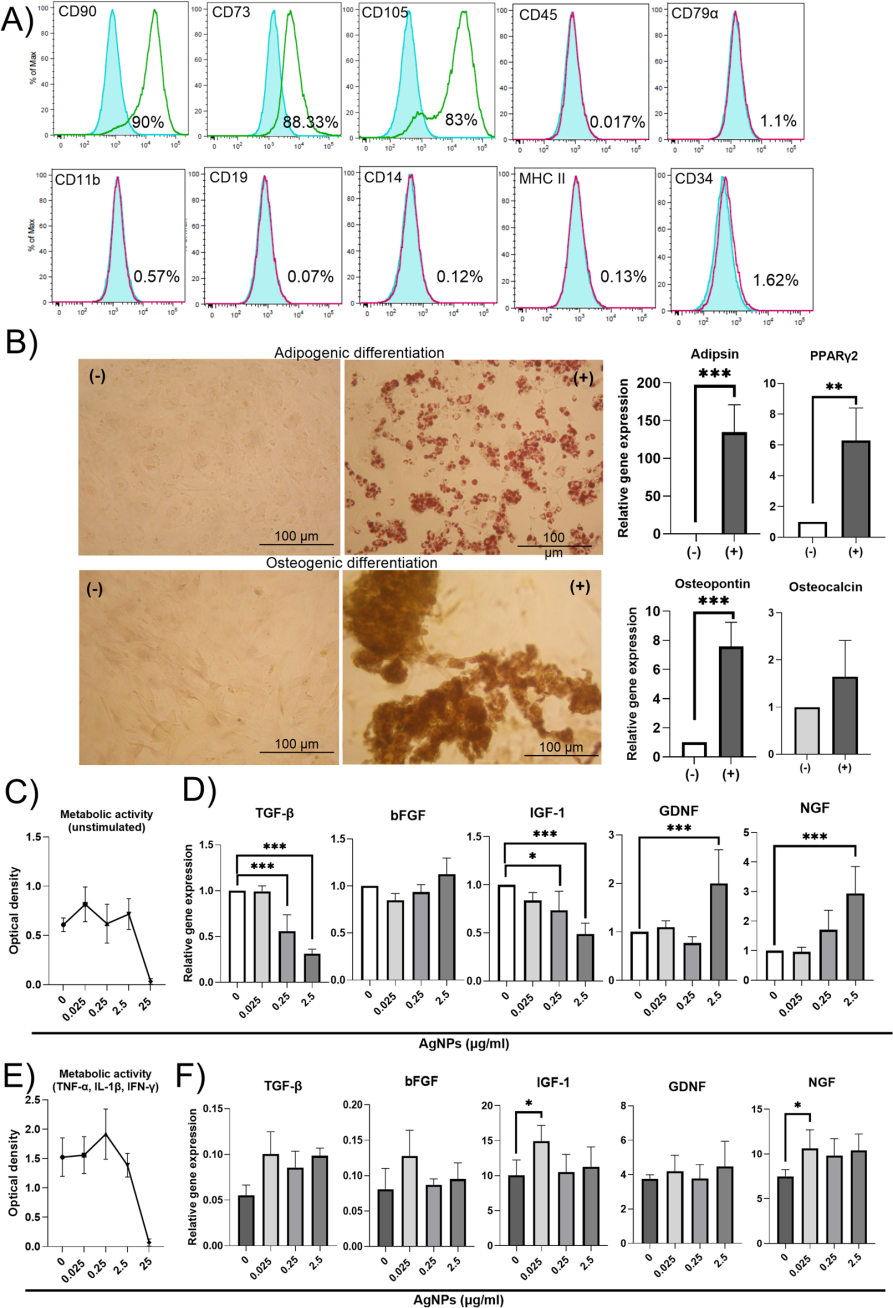
The in vitro effect of AgNPs on the viability and gene expression of MSCs. (**A**) MSCs were characterized by flow cytometry. Representative histograms show the presence of CD90, CD73 and CD105 and the absence of CD45, CD79α, CD11b, CD19, CD14, CD34 and MHCII markers on MSCs. The percentage of cell markers represent the mean from 3 different experiments. (**B**) Representative images of MSCs stained with Oil Red O (adipogenic differentiation) or Alizarin Red S (osteogenic differentiation) and RT-PCR analysis of adipsin, PPARγ2, osteopontin and osteocalcin show the potential of cells to differentiate into adipogenic and osteogenic cell lines. (**C**) MSCs were cultivated (unstimulated) in the presence of different concentrations of AgNPs (0–25 µg/ml) and metabolic activity was tested by WST-1 assay on day 7 of cultivation. (**D**) MSCs were cultivated (unstimulated) with a selected concentration of AgNPs (0–2.5 µg/ml). The expression of genes for TGF-β, bFGF, IGF-1, GDNF and NGF was analyzed by RT-PCR on day 7 of cultivation. (**E**) MSCs were cultivated in the presence of different concentrations of AgNPs (0–25 µg/ml). The cells were stimulated with proinflammatory cytokines TNF-α, IL-1β and IFN-γ for 48 h before the end of cultivation. Metabolic activity was determined on day 7 of cultivation by WST-1 assay. (**F**) MSCs were cultivated in the presence of selected concentrations of AgNPs (0–2.5 µg/ml). The cells were stimulated with proinflammatory cytokines TNF-α, IL-1β and IFN-γ for 48 h before the end of cultivation. The expression of genes for TGF-β, bFGF, IGF-1, GDNF and NGF was analyzed by RT-PCR on day 7 of cultivation. The data were normalized according to an unstimulated control sample. Each bar represents the mean + SD from three independent experiments. An asterisk represents significant differences (*p < 0.05; ***p < 0.001).

MSCs isolated from adipose tissue were characterized using flow cytometry. As is shown in (Fig. 1A), the cells prepared according to our standard protocol were negative for leucocyte markers CD45, CD79α, CD11b, CD19, CD14, CD34 and MHC II and positive for CD73, CD90 and CD105 (the percentage of cell markers represent the mean from 3 different batches of MSCs). The cells were able to differentiate into adipocytes and osteoblasts, as is shown in representative images and RT-PCR analysis (Fig. 1B). The cells cultivated under specific differentiation media significantly increased the expression of genes for adipsin, PPARγ2 and osteopontin and elevated the expression of gene for osteocalcin. As described previously, the cells were adherent to the plastic surfaces^42^.

### The in vitro effect of AgNPs on the viability and gene expression of MSCs

It was shown that none of the tested low concentrations of AgNPs, except for 25 µg/ml, caused decreased metabolic activity of MSCs compared to the untreated control cells (Fig. 1C). Since the concentration of 25 µg/ml decreased the metabolic activity of MSCs to a minimum, we assumed that this dose was already harmful for the cells and for this reason, it was excluded from the following test. Interestingly, the metabolic activity of unstimulated MSCs increased with the lowest concentration (0.025 µg/ml) of NPs. On contrary, stimulated MSCs increased metabolic activity under concentration of 0.25 µg/ml. According to the RT-PCR analysis, the concentrations of 2.5 and 0.25 µg/ml of AgNPs significantly decreased the expression of genes for TGF-β and IGF-1 while the concentration of 2.5 µg/ml significantly increased the expression of genes for NGF and GDNF in unstimulated MSCs. There was also observed an increase in the expression of gene for bFGF (with concentration of 2.5 µg/ml), however, this change was not significant. On the contrary, the concentration of 0.025 µg/ml had no significant effect on the expression of the tested genes (Fig. 1D). To test the ability of MSCs cultivated with AgNPs to react to cytokine stimulation, MSCs were stimulated for the last 48 h of cultivation with TNF-α, IL-1β and IFN-γ. As in the previous experiment, 25 µg/ml of AgNPs caused decreased metabolic activity of MSCs compared to the untreated control cells (Fig. 1E). On contrary, the expression of the gene for TGF-β and NGF was increased by all tested concentrations of AgNPs. Moreover, the concentration of 0.025 µg/ml of AgNPs increased the expression of genes for IGF-1 and bFGF compared to the control (Fig. 1F).

### The in vitro effect of MSCs or MSCs/AgNPs on gene expression in degenerated retina

**Fig. 2:**
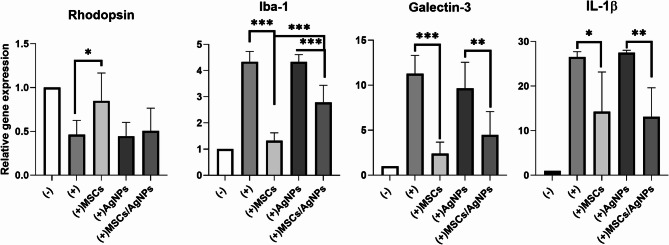
The in vitro effects of MSCs and MSCs cultivated with AgNPs on the expression of genes in the degenerated retina. Retinas isolated from eyes with degenerated retinas were cultivated alone (+), in the presence of MSCs ((+)MSCs), in the presence of 0.025 µg/ml of AgNPs ((+)AgNPs) or in the presence of MSCs cultivated with AgNPs ((+)MSCs/AgNPs). The expression of genes for rhodopsin, Iba-1, galectin-3 and IL-1β was determined in the retina after 48-h cultivation. The cultivated retina from a healthy mouse was used as a negative control (−). Each bar represents the mean + SD from three independent experiments. An asterisk represents significant differences (*p < 0.05; **p < 0.01; ***p < 0.001).

The degeneration of retina was induced by the repeated (3 times in total) intraperitoneal applications of NaIO_3_. This procedure related to the decreased expression of *RPE-65* and *Rlbp* genes in the posterior segment (unpublished results) and the decreased expression of gene for rhodopsin in the retina, indicating the reduced function or death of photoreceptor cells. On the contrary, the number of CD45 and CD11b positive cells increased in the NaIO_3_-treated retina. This effect was associated with the increased expression of genes for Iba-1 (microglia/macrophages marker), IL-1β (proinflammatory factor) and galectin-3 (lectin associated with the induction of neuroinflammation). To test the immunomodulatory and regenerative potential of MSCs or MSCs/AgNPs, the retinas isolated from the eyes of NaIO_3_-treated mice were added into wells with MSCs or MSCs cultivated for 5 days with AgNPs (the dose of AgNPs was selected according to in vitro experiments and our previous in vivo study^29^). As it is shown on Fig. 2, the presence of untreated MSCs increased the expression of the gene for rhodopsin and decreased the expression of genes for Iba-1, galectin-3 and IL-1β in degenerated retinas, indicating the regenerative and immunoregulatory potential of MSCs on the retinal cells. On the other hand, MSCs cultivated with AgNPs exert lower effect in the decreased of the *Iba-1* and *galectin-3* expression and did not increase the expression of the gene for rhodopsin in degenerated retinas. There was also a significant difference between the expression of the gene for Iba-1 in retinas cultivated in the presence of MSCs and MSCs pretreated with AgNPs. However, the regulation of the *IL-1β* gene was more significant in the retinas treated with the combination of MSCs and AgNPs. These results suggest that AgNPs decrease the immunomodulatory and regenerative properties of MSCs in vitro.

### Detection of labeled MSCs in degenerated retina

**Fig. 3:**
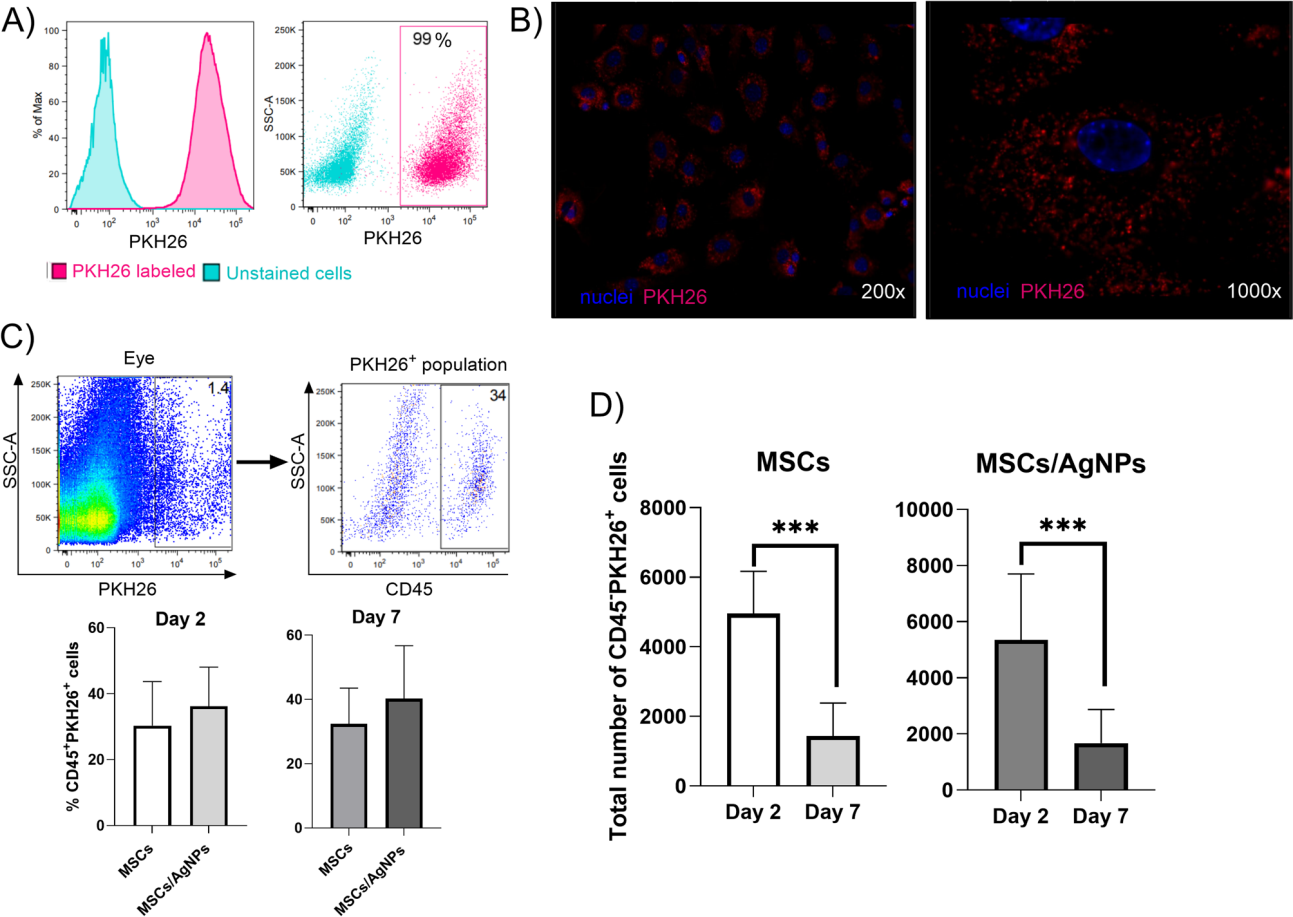
Detection of labeled MSCs in the posterior segment. The intensity and quality of PKH26 labeling are shown on (**A**) representative histogram and dot plot from flow cytometry analysis and on (**B**) representative fluorescence microscopy image (original magnification 200x and 1000x). MSCs (10 000 cells) labeled with vital dye PKH26, were intravitreally injected into the degenerated eye alone (MSCs), or together with 0.025 µg AgNPs (MSCs/AgNPs). The whole posterior segment was analyzed on day 2 or on day 7 after application by flow cytometry. (**C**) Representative dot plots show gating strategy and the percentages of CD45^+^ cells from all PKH26^+^ cells were considered as MSCs phagocyted by immune cells. (**D**) The total number of PKH26-labeled MSCs (CD45^−^PKH26^+^ cells) that persisted in the posterior segment on days 2 and 7. Data represents the mean + SD from three independent experiments. An asterisk represents significant differences (***p < 0.001). The total number of experimental animals in each group were 6 mice for analysis on day 2 and 15 mice for analysis on day 7.

The distribution of PKH26-labeled MSCs and PKH26-labeled MSCs applied with AgNPs (MSCs/AgNPs) was analyzed in the whole posterior segment on days 2 and 7 after intravitreal application. It was observed that PKH26-labeled MSCs possessed a sufficient fluorescence signal detectable by flow cytometry (Fig. 3A) and by fluorescence microscopy (Fig. 3B). The retinal samples were also stained with CD45 antibody to exclude the MSCs phagocyted by immune cells. It was observed that approximately 30–40% of MSCs and MSCs/AgNPs were phagocyted by CD45^+^ cells in both studied times (Fig. 3C). Figure 3D shows that on day 2, approximately 50% (from the total number of 10 000 applied MSCs) of PKH26^+^CD45^−^ cells (MSCs) were present in the eye. However, on day 7, only 15% of applied MSCs were detected. There were no significant differences between MSCs applied alone and MSCs applied with AgNPs. This data suggests that AgNPs have no significant effect on the survival of MSCs in the degenerated retina.

### The effect of MSCs, AgNPs or MSCs/AgNPs on the gene expression and the distribution of the rhodopsin in the degenerated retina

**Fig. 4:**
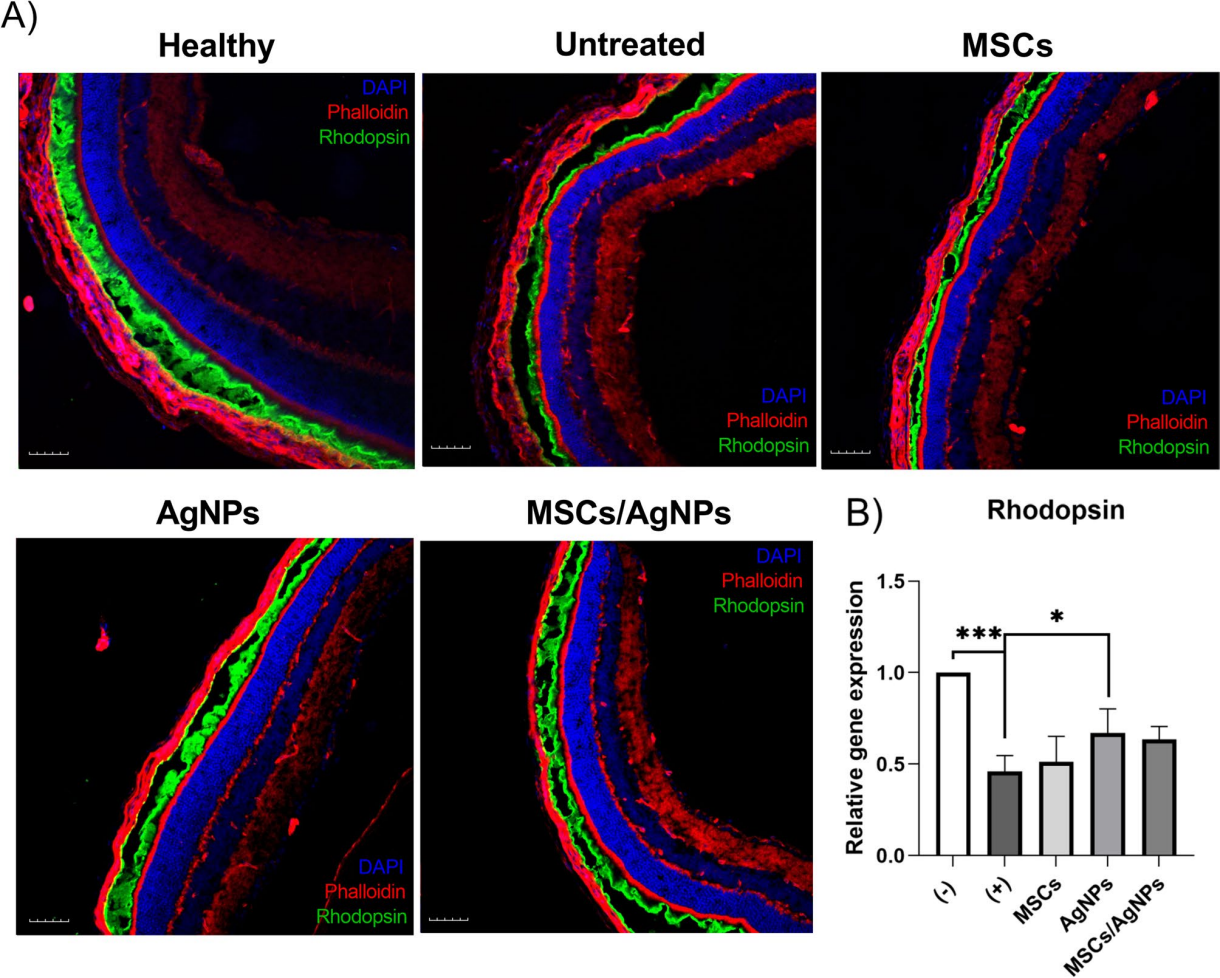
The effect of MSCs, AgNPs or a combination of MSCs with AgNPs on the photoreceptors in the degenerated retina in vivo. MSCs (MSCs), AgNPs (AgNPs) or MSCs with AgNPs (MSCs/AgNPs) were intravitreally injected into the degenerated retina 48 h before the last application of NaIO_3_. Retinas were analyzed on day 7 after application of MSCs, AgNPs or MSCs/AgNPs for (**A**) the presence of rhodopsin in the photoreceptor layer and (**B**) for the expression of genes for rhodopsin. Degenerated retinas treated with only serum free medium (+) were used as a positive control, retinas from healthy mice (−) were used as a negative control. Each bar represents the mean + SD from three independent experiments. An asterisk represents significant differences (*p < 0.05; ***p < 0.001). The total number of experimental animals in each group were 6 mice for RT-PCR analysis and 3 mice for fluorescence microscopy.

Microscopical images showing the degeneration of photoreceptor layer (based on rhodopsin staining of the frozen section of the eye) in the retinas after the repeated application of NaIO_3_ (Fig. 4A). This effect was confirmed by RT-PCR (Fig. 4B) by significant decrease in the expression of the gene for rhodopsin in the NaIO_3_ retinas compared to healthy control sample (negative control). The application of MSCs or MSCs/AgNPs did not show improvement in the *rhodopsin* expression, however we have observed a significant increase in the AgNP-treated group.

### The effect of MSCs, AgNPs or MSCs/AgNPs on gene expression and the presence of the microglia/macrophage population in the degenerated retina

**Fig. 5:**
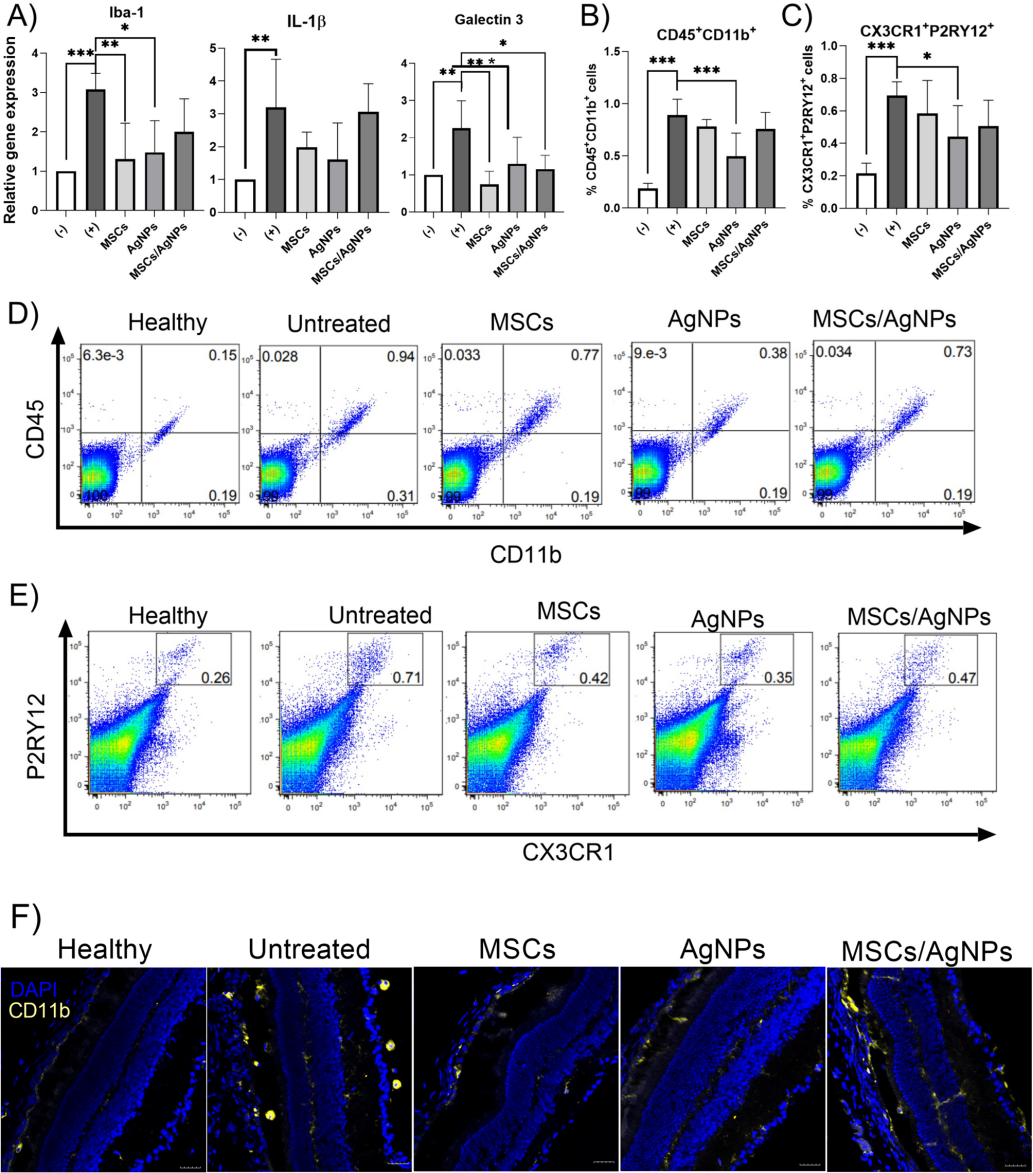
The effect of MSCs, AgNPs or a combination of MSCs with AgNPs on the immune cells in the degenerated retina in vivo. MSCs (MSCs), AgNPs (AgNPs) or MSCs with AgNPs (MSCs/AgNPs) were intravitreally injected into the degenerated retina 48 h before the last application of NaIO_3_. Retinas were analyzed on day 7 after application of MSCs, AgNPs or MSCs/AgNPs for (**A**) the expression of genes for Iba-1, IL-1β and galectin-3, (**B**) for the infiltration of CD45^+^CD11b^+^ cells and (**C**) for the infiltration of CX3CR1^+^P2RY12^+^ cells. Representative dot plots show the infiltration of (**D**) CD45^+^CD11b^+^ cells and (**D**) infiltration of (**E**) CX3CR1^+^P2RY12^+^ cells. (**F**) Fluorescence microscopy images show the distribution of CD11b^+^ cells in the retinal sections. Degenerated retinas treated with only serum free medium (+) were used as a positive control, retinas from healthy mice (−) were used as a negative control. Each bar represents the mean + SD from three independent experiments. An asterisk represents significant differences (*p < 0.05; **p < 0.01; ***p < 0.001). The total number of experimental animals in each group were 6 mice for RT-PCR, 6 mice for flow cytometry and 3 mice for fluorescence microscopy.

Repeated application of NaIO_3_ significantly increased the expression of genes for Iba-1, galectin-3 and IL-1β compared to healthy untreated control (negative control). In addition, an enhanced presence of CD45^+^CD11b^+^ cells (microglia/macrophages) and CX3CR1^+^P2RY12^+^ cells (microglia) was observed in the degenerated retina. Mice with degenerated retina received (into the right eye) MSCs, 0.025 µg of AgNPs or a combination of MSCs/AgNPs, and retinas were tested on day 7 by RT-PCR for the expression of selected genes (Fig. 5A) or by flow cytometry for the presence of microglia/macrophage markers (Fig. 5B–E). It was observed that in all treated groups, the local expression of gene for galectin-3 was significantly decreased compared to degenerated retina treated with the serum free medium (untreated control). Moreover, the application of MSCs or AgNPs decreased the expression of genes for Iba-1 and IL-1β (compared to untreated control), however the combined therapy reduced the effect on the changes in the expression of these genes (Fig. 5A). Flow cytometry analysis of retinas showed that in all treated groups the number of CD45^+^CD11b^+^ cells (Fig. 5B) and CX3CR1^+^P2RY12^+^ cells (Fig. 5C) slightly decreased in comparison with the positive control treated with the serum free medium. However, the decrease was only significant in the AgNP-treated group. Figure 5F show the distribution of the CD11b + cells, as a marker for monocytes/macrophages and microglia, in the healthy, degenerated and MSC-, AgNP- or MSC/AgNP-treated retinas.

### Gene expression analysis of CD45^+^ cells sorted from MSCs- or MSCs/AgNP-treated degenerated retina

**Fig. 6:**
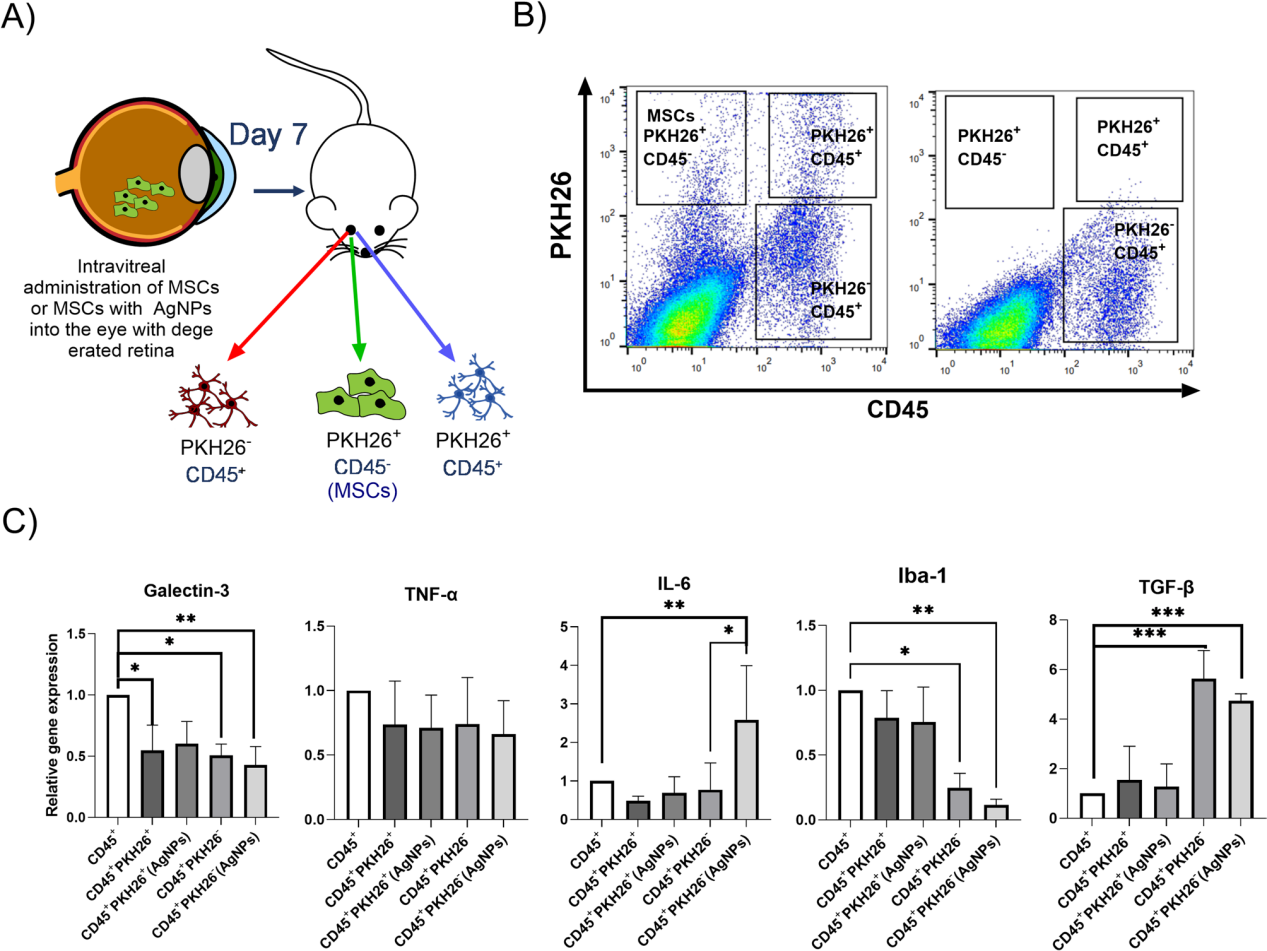
The effect of MSCs or MSCs applied with AgNPs (AgNPs) treatment on the gene expression of CD45^+^ populations sorted from the degenerate retina. (**A**) Schema of the experiment: MSCs were labeled with vital dye PKH26 and intravitreally injected into the eye with the degenerated retina induced by the application of NaIO_3_. Cells from the retina were sorted into 3 populations on day 7 after application. (**B**) The representative dot plot shows the PKH26^+^CD45^−^ population characterized as MSCs, the PKH26^+^CD45^+^ population characterized as immune cells with phagocyted MSCs and the PKH26^−^CD45^+^ population characterized as immune cells without phagocyted MSCs. (**C**) Populations of CD45^+^ cells sorted from the retina were analyzed by RT-PCR for the expression of genes for galectin-3, TNF-α, IL-6, Iba-1 and TGF-β. CD45^+^ cells sorted from the untreated degenerated retina (CD45^+^) were used as a control. Each bar represents the mean + SD from three independent experiments. An asterisk represents significant differences (*p < 0.05; **p < 0.01; ***p < 0.001). The total numbers of experimental animals used for the separation of the PKH26^+^CD45^−^, PKH26^+^CD45^+^ and PKH26^−^CD45^+^ populations were 8 for the MSC-treated group, 8 for the MSC/AgNP-treated group and 4 for the untreated group. Due to the small amount of separated PKH26^−^ cells, the final analysis was provided by combining samples from 2 mice into 1 sample sufficient for obtaining an adequate amount of mRNA.

Cell suspension from the MSCs- or MSCs/AgNPs-treated degenerated retinas was sorted into 3 populations (Fig. 6A,B). The population positive for PKH26 and the population negative for CD45 were considered as MSCs. Populations positive for CD45 were divided according to the positivity for PKH26. Double positive cells (CD45^+^PKH26^+^) were considered as immune cells with phagocyted MSCs. CD45 positive cells negative for PKH26 were considered as immune cells (microglia or macrophages) without phagocyted MSCs. Sorted PKH26^+^CD45^+^ and PKH26^−^CD45^+^ cells were analyzed for the expression of genes for galectin-3, TNF-α, IL-6, Iba-1 and TGF-β and compared with the CD45^+^ population from the untreated degenerated retina (Fig. 6C). A decreased expression of the gene for galectin-3 was present in all CD45^+^ cells in both treated groups. We did not observe any significant changes in the expression of the gene for TNF-α between groups, however the expression of the *IL-6* gene was increased in CD45^+^ cells in the MSCs/AgNPs treated group. In addition, CD45^+^PKH26^−^ cells in both groups increased the expression of the gene for TGF-β and decreased the expression of the gene for Iba-1.

### Gene expression analysis of MSCs sorted from MSCs- or MSC/AgNP-treated degenerated retina

**Fig. 7:**
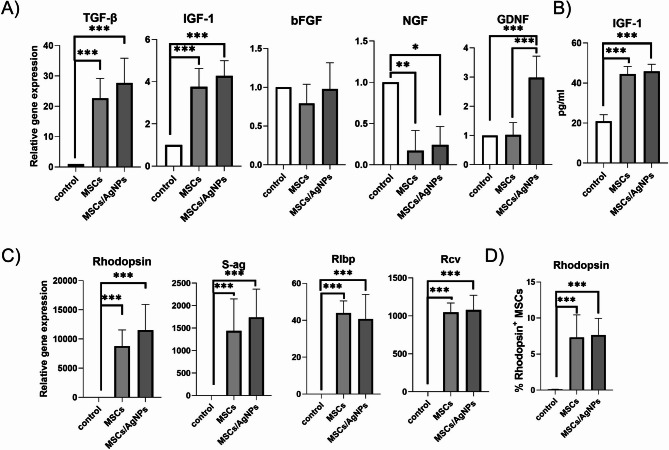
The effects of the degenerated retina on changes in the expression of genes in MSCs. PKH26^+^CD45^−^ cells (MSCs), sorted from the retina on day 7 after the application, were analyzed for the expression of genes for (**A**) growth factors and for (**B**) for the production of IGF-1, (**C**) for the expression of retinal markers and for the (**D**) rhodopsin levels. MSCs were applied alone (MSCs) or with AgNPs (MSCs/AgNPs). Naïve untreated MSCs were used as a control (control). Each bar represents the mean + SD from three independent experiments. An asterisk represents significant differences (*p < 0.05; **p < 0.01; ***p < 0.001). For analysis were used PKH26^+^ MSCs obtained from the same animals used for sorting of CD45^+^ population.

To examine the effect of the degenerated retinal environment on MSCs, and the possible changes between MSCs and MSCs/AgNPs, CD45^−^PKH26^+^ cells sorted from the retina were analyzed for the expression of genes for immunomodulatory and growth factors (Fig. 7A), for the production of IGF-1 (Fig. 7B) and for markers of retinal cells (Fig. 7C). As shown in Fig. 7A, MSCs sorted from both treated groups increased the expression of genes for TGF-β and for IGF-1 compared to untreated naïve cells. The increase of IGF-1 was also confirmed on protein level (Fig. 7B). Interestingly, the expression of the gene for GDNF was significantly increased only in the MSC/AgNP treated group. On the contrary, the expression of the gene for NGF was decreased in both tested groups. It was also observed that MSCs and MSCs/AgNPs separated from the retina significantly increased the expression of genes for retinal cell markers such as rhodopsin, S-antigen (S-ag), retinaldehyde binding protein 1 (Rlbp) and recoverin (Rcv) (Fig. 7C). The differentiation potential of MSCs was also confirmed on the protein level by flow cytometry analysis. Figure 7D shows the increased presence of rhodopsin in the MSCs separated from the retinas. Similarly, as in the RT-PCR analysis, the cells applied in the presence of AgNPs did not show any significant differences in production of rhodopsin compared to MSC injected alone.

## Discussion

Upregulation of the immune response represents one of the main causes of undesirable changes in retinal diseases in general. Major immune cell populations in the retina include microglia, which represent a population of resident macrophages. Microglia, in their physiological state, are the cells responsible for maintaining homeostasis and the production of several factors such as NGF, GDNF and bFGF^21,22^. However, activated microglia and macrophages accumulate in the degenerated retinal tissue, produce proinflammatory factors and contribute to the formation of vascularization. It has been shown that microglia and macrophages are responsible for the production of cytokines from the IL-1 family and amplify the immune reaction in the eye^23,24,27^. Activated microglia also express higher levels of galectin-3, which is associated with neurotoxicity^30^. Therefore, focusing on the regulation of immune cells in the retina represents a promising target for potential therapy.

To date, there are several genetically and pharmaceutically induced experimental mouse models of retinal degeneration^1^, which mimic the condition in the diseased retina such as AMD and DR. One of the possibilities to generate retinal degeneration resembling AMD in the mouse is the application of NaIO_3_. Intraperitoneal or intravenous administration of NaIO_3_ represents a relatively easily reproducible experimental model in which degeneration of photoreceptors occurs, and the extent of the damage is dependent on the applied doses^43–45^. Moreover, it was shown that the destruction of retinal cells by NaIO_3_ is associated with a local accumulation of immune cells^43^. In the current study, we established a chronic model of retinal degeneration induced by repeated application of low doses of NaIO_3_ which caused a decreased expression of the gene for rhodopsin, but increased expression of the genes for Iba-1, IL-1β and galectin-3 and an increased number of CD45^+^CD11b^+^ (macrophages/microglia markers) and CX3CR1^+^P2RY12^+^ (the specific combination of microglia markers) cells in the neuroretina compared to the healthy eye. It was also observed that the expression of *RPE-65* and *Rlbp* genes decreased in the posterior segment, indicating the reduction of function or damage of RPE cells (unpublished results). Repeated applications of NaIO_3_ also caused a more significant increase in the accumulation of microglia/macrophages compared to a single application (data not shown) and thus provides a useful model for the study of retinal degeneration. In addition, we did not observe any changes in behavior or in the body weight of mice during the repeated applications of NaIO_3_.

NPs are tested in the field of medicine for their unique properties, such as their antibacterial and anti-inflammatory effects. Due to their small size (under 100 nm), NPs can also be used as carriers for various types of drugs, diagnostic markers or as a tracking agent^9^. In general, the effects of NPs are dependent on their sources (natural or synthetic) and their size. It has been reported that smaller sizes have a higher toxic effect on cell lines^38^. This could be caused by the more abundant penetration of smaller NPs into the cells. Several studies have focused on the use of various types of NPs in the treatment of retinal diseases and the beneficial effect of used NPs was mainly associated with their anti-inflammatory and anti-angiogenetic properties^33,35,36^. In our previous study^37^ we observed that AgNPs modulate the immune response in the proinflammatory retinal environment. The downregulation of the neuroinflammation by applications of NPs represents a promising direction in the attenuation of the development of degenerative retinal diseases, yet there is need for a regenerative aspect of the resulting therapy. The application of MSCs has been reported as a potential treatment increasing the survival and regeneration of retinal cells^1,56,57^. While MSCs possess several immunomodulatory properties, it has also been reported that their local application could cause an increased accumulation of microglia or macrophages^46^. Thus, the combination of NP-based therapy, as an antiangiogenic and anti-inflammatory agent and MSC-based therapy as a regenerative and neuroprotective agent could result in optimal treatment. The use of NPs has also been considered as a delivery vehicle for the reprogramming of stem cells^47^. However, some studies reported a negative influence of NPs on MSCs^39,40,43^. Therefore, we examined the potential effects of metal NPs on the immunomodulatory and regenerative properties of MSCs. Although it has been shown that low doses of AgNPs have a relatively weak effect on the characteristic and functional properties of MSCs^40,48^, the potential risk of using therapy combining MSCs with NPs should be taken into consideration.

MSCs are widely studied in various diseases due to their immunomodulatory and regenerative properties. It has been shown that MSCs spontaneously produce TGF-β (potent immunomodulatory factor) and HGF (growth factor), and under proinflammatory conditions upregulate the expression of various immunoregulatory factors such as IDO, PD-L1 (regulation of T lymphocytes), Cox-2 (regulation of B lymphocytes), TSG-6 (modulation of M1/M2 response), and several growth factors such as NGF, GDNF and IGF-1. On the contrary, the expression of genes for TGF-β and HGF in MSCs is downregulated after stimulation with proinflammatory cytokines^2,3,6,7^. In general, MSCs react to the environment on the level of regulation of gene expression, and the spectrum of produced factors is dependent on stimulation^6,7^. For example, we observed that MSCs treated with the supernatant obtained after cultivation of the degenerated retina increased the expression of genes for TGF-β and Cox-2 and decreased the expression of genes for HGF (unpublished results). In this study, intravitreally applied MSCs exerted an increased expression of genes for TGF-β and IGF-1 but had decreased expression of the gene for NGF. Interestingly, we also observed increased expression of the gene for GDNF but a significant value was present only in the MSCs applied with AgNPs. The role of GDNF in the protection and regeneration of retinal cells has been observed in several studies such as optical nerve crush, rhodopsin knockout mice or retinal degeneration mouse model^49–51^. It was also suggested that the protection of photoreceptors by GDNF is mediated by the effects on Müller cells^51^. We have shown that the expression of the gene for TGF-β (a cytokine known as a potent immunomodulatory factor) was increased in the CD45^+^ cells separated from the MSCs- or MSC/AgNP-treated retina. However, a significant upregulation was observed only in the cells without phagocyted MSCs. This cell population also had decreased expression of *Iba-1*, which is a marker for activated microglia and macrophages. Since we used the whole posterior segment for this type of experiment, the tested CD45^+^cells could also include regulatory T-cells (Tregs) or other retinal cell types expressing CD45. For example, it was shown that RPE cells express markers of leukocytes such as CD45^52^. Moreover, RPE cells and Tregs are also able to produce TGF-β^53^, which was significantly increased in our tested CD45^+^ population. In addition, CD45^+^ cells sorted from the retina after the administration of MSCs/AgNPs had increased expression of the gene for IL-6. As IL-6 is a factor which is involved in immune reaction in the retina^54,55^, this increase could play a role in the limited effect of MSC/AgNP therapy. TGF-β and IL-6 are also known as key players in the Treg and Th17 switch and therefore this combination could promote decreased immunomodulatory function of MSC/AgNP combined therapy.

In addition to their immunomodulatory and neuroprotective properties, MSCs can also differentiate into specialized cell types^5^, which could replace the degenerated cells in diseased tissue. It has been demonstrated that MSCs possess the ability to differentiate into cells expressing retinal cell markers, and this effect was enhanced under proinflammatory stimulation^4^. In our study, naïve untreated MSCs expressed minimal levels of retinal gene markers such as *rhodopsin*, *S-ag*, *Rlbp* or *Rcv*, however MSCs separated from the degenerated retina had significantly upregulated expression of all these genes. These results indicate the potential of applied MSCs to differentiate into retina-like cell types.

MSCs have been considered for the treatment of ocular diseases in several studies such as retinal degeneration, corneal inflammation, retinitis pigmentosa^1,2,56–59^. It was reported that intravitreal application of MSCs regulates the immune response in the inflammatory retinal environment^3^. In our experimental model of chronic retinal degeneration, MSCs modulated the expression of *Iba-1*, *galectin-3* and *IL-1β* under both ex vivo and in vivo conditions. In addition, cultivation of degenerated retina with MSCs significantly upregulated the expression of the gene for rhodopsin in the retinal tissue. Considering that the retina was separated from MSCs by the semipermeable membrane, this result indicates the importance of the paracrine action of MSCs. This was in accordance with a previous study performed on damaged cornea^2^. However, MSCs cultivated with AgNPs have a decreased effect on the expression of genes for Iba-1 and rhodopsin in the degenerated retina and similar results were observed in in vivo experiments.

In a recent study, we showed that the long-term cultivation of MSCs in the presence of low doses of AgNPs influences the expression of genes for several immunomodulatory and growth factors. For example, the expression of genes for TGF-β and IGF-1 was significantly decreased in the presence of AgNPs in concentrations of 0.25 and 2.5 µg/ml. On the contrary, the concentration of 2.5 µg/ml of AgNPs upregulated the expression of genes for NGF and GDNF in MSCs. A similar trend was observed in vivo in MSCs separated from degenerated retina. MSCs applied with AgNPs expressed higher levels of genes for NGF and GDNF compared to the cells applied alone. The production of NGF and GDNF by transplanted MSCs has been suggested as an important player in retinal cell regeneration^46,60,61^. From this viewpoint, the co-applications of MSCs with NPs could represent a potential strategy for the enhancement of cell-based therapy. We also observed that the application of AgNPs did not influence the number of detected MSCs in the retina after their intravitreal injection. In accordance with our previous study^37^, the administration of AgNPs alone decreased the expression of genes for Iba-1, IL-1β and galectin-3 in the degenerated retina, similarly to the MSC-only treated group. The results suggest that the negative effects of AgNPs on MSC-based treatment are more likely dependent on the regulatory function on MSCs than possible toxicity. Moreover, the application of AgNPs as a single therapy significantly increased the expression of the gene for rhodopsin in the degenerated retina compared to the untreated degenerated eye. On the contrary, AgNPs decreased the number of CD45^+^CD11b^+^ (microglia/macrophages) and CX3CR1^+^P2RY12^+^ (microglia) cells in the diseased tissue. These results are in accordance with Moriguchi et al.^43^ who described that depletion of macrophages protects photoreceptors from degeneration induced by NaIO_3_.

## Conclusion

In conclusion, our study investigates the possible therapeutic option of MSC-based and NP-based therapy and their combination in the regulation of immune cells which infiltrate the degenerated retina. Local applications of MSCs or AgNPs, as a single therapy, were able to modulate the immune reaction in the experimental model of chronic retinal degeneration. However, the combined applications decreased the immunomodulatory effects of MSCs or AgNPs. Although it was shown that injections of MSCs with the AgNPs did not affect the migration and survival of transplanted cells in the retina, the resulting treatment did not decrease the expression of genes for Iba-1 and IL-1β and the local number of microglia/macrophages. One of the possible reasons for this negative effect could be the triggering of CD45^+^ cells by AgNPs to increase the expression of genes for IL-6, which could interact directly with MSCs or with other cytokines produced by these cells (e.g. TGF-β). Nevertheless, the MSCs applied with AgNPs had increased expression of gene for NGF and GDNF compared to cells injected alone. These results were confirmed in vitro and highlight that the combination of MSCs with NPs could provide the possibility to increase the production of neuroprotective factors in cell-based therapy. However, further examinations are needed to assess the optimal treatment.

## Data Availability

The experimental data that support the findings of this study are available at 10.5281/zenodo.13767702.

## References

[1] Holan, V., Palacka, K. & Hermankova, B. Mesenchymal stem cell-based therapy for retinal degenerative diseases: experimental models and clinical trials. *Cells* **10** (3), 588. 10.3390/cells10030588 (2021). 33799995 10.3390/cells10030588 PMC8001847

[2] Kossl, J. et al. Antiapoptotic properties of mesenchymal stem cells in a mouse model of corneal inflammation. *Stem. Cells. Dev.* **30** (8), 418–427. 10.1089/scd.2020.0195 (2021). 33607933 10.1089/scd.2020.0195

[3] Hermankova, B. et al. The Immunomodulatory potential of mesenchymal stem cells in a retinal inflammatory environment. *Stem Cell. Reviews Rep.* **15** (6), 880–891. 10.1007/s12015-019-09908-0 (2019). 10.1007/s12015-019-09908-0 31863334

[4] Hermankova, B. et al. The identification of interferon-γ as a key supportive factor for retinal differentiation of murine mesenchymal stem cells. *Stem. Cells. Dev.* **26** (19), 1399–1408. 10.1089/scd.2017.0111 (2017). 28728472 10.1089/scd.2017.0111

[5] Salehi, H., Amirpour, N., Razavi, S., Esfandiari, E. & Zavar, R. Overview of retinal differentiation potential of mesenchymal stem cells: A promising approach for retinal cell therapy. *Annals Anat. = Anatomischer Anzeiger: Official Organ. Anatomische Gesellschaft*. **210**, 52–63. 10.1016/j.aanat.2016.11.010 (2017). 10.1016/j.aanat.2016.11.010 27986614

[6] Holan, V. et al. Distinct immunoregulatory mechanisms in mesenchymal stem cells: role of the cytokine environment. *Stem Cell. Reviews Rep.* **12** (6), 654–663. 10.1007/s12015-016-9688-y (2016). 10.1007/s12015-016-9688-y 27665290

[7] Broekman, W. et al. TNF-α and IL-1β-activated human mesenchymal stromal cells increase airway epithelial wound healing in vitro via activation of the epidermal growth factor receptor. *Respir. Res.* **17**, 3. 10.1186/s12931-015-0316-1 (2016). 26753875 10.1186/s12931-015-0316-1 PMC4710048

[8] de Araújo Farias, V., Carrillo-Gálvez, A. B., Martín, F. & Anderson, P. TGF-β and mesenchymal stromal cells in regenerative medicine, autoimmunity and cancer. *Cytokine Growth Factor Rev.* **43**, 25–37. 10.1016/j.cytogfr.2018.06.002 (2018). 29954665 10.1016/j.cytogfr.2018.06.002

[9] Klębowski, B., Depciuch, J., Parlińska-Wojtan, M. & Baran, J. Applications of noble metal-based nanoparticles in medicine. *Int. J. Mol. Sci.* **19** (12), 4031. 10.3390/ijms19124031 (2018). 30551592 10.3390/ijms19124031 PMC6320918

[10] Lappas, C. M. The Immunomodulatory effects of titanium dioxide and silver nanoparticles. *Food Chem. Toxicology: Int. J. Published Br. Industrial Biol. Res. Association*. **85**, 78–83. 10.1016/j.fct.2015.05.015 (2015). 10.1016/j.fct.2015.05.015 26051351

[11] Vuković, B. et al. In vitro study on the Immunomodulatory effects of differently functionalized silver nanoparticles on human peripheral blood mononuclear cells. *J. Biol. Inorg. Chemistry: JBIC : Publication Soc. Biol. Inorg. Chem.* **26** (7), 817–831. 10.1007/s00775-021-01898-0 (2021). 10.1007/s00775-021-01898-0 PMC8412400 34476609

[12] Mohammapdour, R. & Ghandehari, H. Mechanisms of immune response to inorganic nanoparticles and their degradation products. *Adv. Drug Deliv. Rev.* **Jan;180**, 114022. 10.1016/j.addr.2021.114022 (2022). Epub 2021 Nov 2. PMID: 34740764; PMCID: PMC8898339. 34740764 10.1016/j.addr.2021.114022 PMC8898339

[13] Stahl, A. The diagnosis and treatment of age-related macular degeneration. *Deutsches Ärzteblatt Int.* **117** (29–30), 513–520. 10.3238/arztebl.2020.0513 (2020). 10.3238/arztebl.2020.0513 PMC7588619 33087239

[14] Chauhan, M. Z., Rather, P. A., Samarah, S. M., Elhusseiny, A. M. & Sallam, A. B. Current and novel therapeutic approaches for treatment of diabetic macular edema. *Cells* **11** (12), 1950 10.3390/cells11121950 (2022). 10.3390/cells11121950 PMC9221813 35741079

[15] Friedman, D. S. et al. Prevalence of age-related macular degeneration in the united States. *Archives Ophthalmol. (Chicago Ill. : 1960)*. **122** (4), 564–572. 10.1001/archopht.122.4.564 (2004). 10.1001/archopht.122.4.564 15078675

[16] Ying, G. S., Maguire, M. G. & Complications of Age-related Macular Degeneration Prevention Trial Research Group. Development of a risk score for geographic atrophy in complications of the age-related macular degeneration prevention trial. *Ophthalmology* **118** (2), 332–338. 10.1016/j.ophtha.2010.06.030 (2011). 20801521 10.1016/j.ophtha.2010.06.030 PMC2994966

[17] Thomas, C. J., Mirza, R. G. & Gill, M. K. Age-Related macular degeneration. *Med. Clin. N. Am.* **105** (3), 473–491. 10.1016/j.mcna.2021.01.003 (2021). 33926642 10.1016/j.mcna.2021.01.003

[18] Biesemeier, A., Taubitz, T., Julien, S., Yoeruek, E. & Schraermeyer, U. Choriocapillaris breakdown precedes retinal degeneration in age-related macular degeneration. *Neurobiol. Aging*. **35** (11), 2562–2573. 10.1016/j.neurobiolaging.2014.05.003 (2014). 24925811 10.1016/j.neurobiolaging.2014.05.003

[19] Curcio, C. A., Medeiros, N. E. & Millican, C. L. Photoreceptor loss in age-related macular degeneration. *Investig. Ophthalmol. Vis. Sci.* **37** (7), 1236–1249 (1996). 8641827

[20] Dunaief, J. L., Dentchev, T., Ying, G. S. & Milam, A. H. The role of apoptosis in age-related macular degeneration. *Archives Ophthalmol. (Chicago Ill. : 1960)*. **120** (11), 1435–1442. 10.1001/archopht.120.11.1435 (2002). 10.1001/archopht.120.11.1435 12427055

[21] Madeira, M. H., Boia, R., Santos, P. F., Ambrósio, A. F. & Santiago, A. R. Contribution of microglia-mediated neuroinflammation to retinal degenerative diseases. *Mediat. Inflamm.* **2015**, 673090 10.1155/2015/673090 (2015). 10.1155/2015/673090 PMC4385698 25873768

[22] Rashid, K., Akhtar-Schaefer, I. & Langmann, T. Microglia in retinal degeneration. *Front. Immunol.* **10**, 1975. 10.3389/fimmu.2019.01975 (2019). 31481963 10.3389/fimmu.2019.01975 PMC6710350

[23] Choi, S., Guo, L. & Cordeiro, M. F. Retinal and brain microglia in multiple sclerosis and neurodegeneration. *Cells* **10** (6), 1507. 10.3390/cells10061507 (2021). 34203793 10.3390/cells10061507 PMC8232741

[24] Altmann, C. & Schmidt, M. H. H. The role of microglia in diabetic retinopathy: inflammation, microvasculature defects and neurodegeneration. *Int. J. Mol. Sci.* **19** (1), 110. 10.3390/ijms19010110 (2018). 29301251 10.3390/ijms19010110 PMC5796059

[25] Kinuthia, U. M., Wolf, A. & Langmann, T. Microglia and inflammatory responses in diabetic retinopathy. *Front. Immunol.* **11**, 564077. 10.3389/fimmu.2020.564077 (2020). 33240260 10.3389/fimmu.2020.564077 PMC7681237

[26] Noailles, A. et al. Persistent inflammatory state after photoreceptor loss in an animal model of retinal degeneration. *Sci. Rep.* **6**, 33356. 10.1038/srep33356 (2016). 27624537 10.1038/srep33356 PMC5022039

[27] Wooff, Y., Man, S. M., Aggio-Bruce, R., Natoli, R. & Fernando, N. IL-1 family members mediate cell death, inflammation and angiogenesis in retinal degenerative diseases. *Front. Immunol.* **10**, 1618. 10.3389/fimmu.2019.01618 (2019). 31379825 10.3389/fimmu.2019.01618 PMC6646526

[28] Liu, Y. et al. Galectin-3 regulates microglial activation and promotes inflammation through TLR4/MyD88/NF-kB in experimental autoimmune uveitis. *Clin. Immunol. (Orlando Fla)*. **236**, 108939. 10.1016/j.clim.2022.108939 (2022). 10.1016/j.clim.2022.108939 35121106

[29] Bauer, P. M. et al. Inflamed in vitro retina: cytotoxic neuroinflammation and galectin-3 expression. *PloS One*. **11** (9), e0161723. 10.1371/journal.pone.0161723 (2016). 27612287 10.1371/journal.pone.0161723 PMC5017668

[30] Mendonça, H. R. et al. Lack of Galectin-3 attenuates neuroinflammation and protects the retina and optic nerve of diabetic mice. *Brain Res.* **1700**, 126–137. 10.1016/j.brainres.2018.07.018 (2018). 30016630 10.1016/j.brainres.2018.07.018

[31] Finocchio, L., Zeppieri, M., Gabai, A., Spadea, L. & Salati, C. Recent advances of Adipose-Tissue-Derived mesenchymal stem Cell-Based therapy for retinal diseases. *J. Clin. Med.* **12** (22), 7015. 10.3390/jcm12227015 (2023). 38002628 10.3390/jcm12227015 PMC10672618

[32] Hermankova, B., Javorkova, E., Palacka, K. & Holan, V. Perspectives and limitations of mesenchymal stem Cell-Based therapy for corneal injuries and retinal diseases. *Cell Transplant.* **34**, 9636897241312798. 10.1177/09636897241312798 (2025). 39856809 10.1177/09636897241312798 PMC11760125

[33] Jo, D. H., Kim, J. H., Yu, Y. S., Lee, T. G. & Kim, J. H. Antiangiogenic effect of silicate nanoparticle on retinal neovascularization induced by vascular endothelial growth factor. *Nanomedicine: Nanatechnol. Biology Med.* **8** (5), 784–791. 10.1016/j.nano.2011.09.003 (2012). 10.1016/j.nano.2011.09.003 21945900

[34] Fiorani, L. et al. Cerium oxide nanoparticles reduce microglial activation and neurodegenerative events in light damaged retina. *PloS One*. **10** (10), e0140387. 10.1371/journal.pone.0140387 (2015). 26469804 10.1371/journal.pone.0140387 PMC4607482

[35] Jo, D. H. et al. Anti-angiogenic effect of bare titanium dioxide nanoparticles on pathologic neovascularization without unbearable toxicity. *Nanomedicine: Nanatechnol. Biology Med.* **10** (5), 1109–1117. 10.1016/j.nano.2014.02.007 (2014). 10.1016/j.nano.2014.02.007 24566275

[36] Song, H. B. et al. Intraocular application of gold nanodisks optically tuned for optical coherence tomography: inhibitory effect on retinal neovascularization without unbearable toxicity. *Nanomedicine: Nanatechnol. Biology Med.* **13** (6), 1901–1911. 10.1016/j.nano.2017.03.016 (2017). 10.1016/j.nano.2017.03.016 28400160

[37] Palacka, K. et al. The Immunomodulatory effect of silver nanoparticles in a retinal inflammatory environment. *Inflammation*10.1007/s10753-024-02128-w (2024). 10.1007/s10753-024-02128-w PMC12234591 39190103

[38] De Matteis, V. & Rizzello, L. Noble metals and soft bio-inspired nanoparticles in retinal diseases treatment: A perspective. *Cells* **9** (3), 679. 10.3390/cells9030679 (2020). 32164376 10.3390/cells9030679 PMC7140625

[39] Holan, V. et al. The impact of metal nanoparticles on the immunoregulatory and therapeutic properties of mesenchymal stem cells. *Stem Cell. Reviews Rep.* **19** (5), 1360–1369. 10.1007/s12015-022-10500-2 (2023). 10.1007/s12015-022-10500-2 36810951

[40] Echalar, B. et al. Effects of antimicrobial metal nanoparticles on characteristics and function properties of mouse mesenchymal stem cells. *Toxicol. In Vitro* 105536 10.1016/j.tiv.2022.105536 (2023). 10.1016/j.tiv.2022.105536 36528116

[41] Brzicova, T. et al. Molecular responses in THP-1 macrophage-like cells exposed to diverse nanoparticles. *Nanomaterials* **9** (5), 687. 10.3390/nano9050687 (2019). 31052583 10.3390/nano9050687 PMC6567235

[42] Svobodova, E. et al. The role of mouse mesenchymal stem cells in differentiation of Naive T-cells into anti-inflammatory regulatory T-cell or Proinflammatory helper T-cell 17 population. *Stem. Cells. Dev.* **21** (6), 901–910. 10.1089/scd.2011.0157 (2012). 21663543 10.1089/scd.2011.0157 PMC3315754

[43] Moriguchi, M. et al. Irreversible photoreceptors and RPE cells damage by intravenous sodium iodate in mice is related to macrophage accumulation. *Investig. Ophthalmol. Vis. Sci.* **59** (8), 3476–3487. 10.1167/iovs.17-23532 (2018). 30025075 10.1167/iovs.17-23532

[44] Balmer, J., Zulliger, R., Roberti, S. & Enzmann, V. Retinal cell death caused by sodium iodate involves multiple caspase-dependent and caspase-independent cell-death pathways. *Int. J. Mol. Sci.* **16** (7), 15086–15103. 10.3390/ijms160715086 (2015). 26151844 10.3390/ijms160715086 PMC4519888

[45] Chowers, G. et al. Course of sodium iodate-induced retinal degeneration in albino and pigmented mice. *Investig. Ophthalmol. Vis. Sci.* **58** (4), 2239–2249. 10.1167/iovs.16-21255 (2017). 28418497 10.1167/iovs.16-21255

[46] Ezquer, M. et al. Intravitreal administration of multipotent mesenchymal stromal cells triggers a cytoprotective microenvironment in the retina of diabetic mice. *Stem Cell Res. Ther.* **7**, 42. 10.1186/s13287-016-0299-y (2016). 26983784 10.1186/s13287-016-0299-y PMC4793534

[47] Liu, W. H. et al. Human induced pluripotent stem cell and nanotechnology-based therapeutics. *Cell Transplant.* **24** (11), 2185–2195. 10.3727/096368914X685113 (2015). 25299513 10.3727/096368914X685113

[48] Rossner, P. Jr et al. Metal nanoparticles with antimicrobial properties: the toxicity response in mouse mesenchymal stem cells. *Toxics* **11** (3), 253. 10.3390/toxics11030253 (2023). 36977018 10.3390/toxics11030253 PMC10057305

[49] Flachsbarth, K. et al. Pronounced synergistic neuroprotective effect of GDNF and CNTF on axotomized retinal ganglion cells in the adult mouse. *Exp. Eye Res.* **176**, 258–265. 10.1016/j.exer.2018.09.006 (2018). 30237104 10.1016/j.exer.2018.09.006

[50] García-Caballero, C. et al. Photoreceptor preservation induced by intravitreal controlled delivery of GDNF and gdnf/melatonin in rhodopsin knockout mice. *Mol. Vis.* **24**, 733–745 (2018). 30581280 PMC6279195

[51] Del Río, P. et al. GDNF-induced osteopontin from Müller glial cells promotes photoreceptor survival in the Pde6brd1 mouse model of retinal degeneration. *Glia* **59** (5), 821–832. 10.1002/glia.21155 (2011). 21360756 10.1002/glia.21155

[52] Limb, G. A. et al. Expression of hematopoietic cell markers by retinal pigment epithelial cells. *Curr. Eye Res.* **16** (10), 985–991. 10.1076/ceyr.16.10.985.9009 (1997). 9330849 10.1076/ceyr.16.10.985.9009

[53] Keino, H., Horie, S. & Sugita, S. Immune privilege and eye-derived T-regulatory cells. *J. Immunol. Res.*10.1155/2018/1679197 (2018). 10.1155/2018/1679197 PMC5985108 29888291

[54] Jo, D. H. et al. Interaction between microglia and retinal pigment epithelial cells determines the integrity of outer blood-retinal barrier in diabetic retinopathy. *Glia* **67** (2), 321–331. 10.1002/glia.23542 (2019). 30444022 10.1002/glia.23542

[55] Rojas, M. et al. Role of IL-6 in angiotensin II-induced retinal vascular inflammation. *Investig. Ophthalmol. Vis. Sci.* **51** (3), 1709–1718. 10.1167/iovs.09-337 (2010). 19834028 10.1167/iovs.09-3375 PMC2868419

[56] An, W. et al. Mesenchymal stem cells and mesenchymal stem cell-derived exosomes: a promising strategy for treating retinal degenerative diseases. *Mol. Med. (Cambridge Mass)*. **31** (1), 75. 10.1186/s10020-025-01120-w (2025). 39984849 10.1186/s10020-025-01120-w PMC11846226

[57] Becherucci, V. et al. The new era of therapeutic strategies for the treatment of retinitis pigmentosa: A narrative review of pathomolecular mechanisms for the development of Cell-Based therapies. *Biomedicines* **11** (10), 2656. 10.3390/biomedicines11102656 (2023). 37893030 10.3390/biomedicines11102656 PMC10604477

[58] Labrador-Velandia, S. et al. Mesenchymal stem cells provide paracrine neuroprotective resources that delay degeneration of co-cultured organotypic neuroretinal cultures. *Exp. Eye Res.* **185**, 107671. 10.1016/j.exer.2019.05.011 (2019). 31108056 10.1016/j.exer.2019.05.011

[59] Jindal, N., Banik, A., Prabhakar, S., Vaiphie, K. & Anand, A. Alteration of neurotrophic factors after transplantation of bone marrow derived Lin-ve stem cell in NMDA-Induced mouse model of retinal degeneration. *J. Cell. Biochem.* **118** (7), 1699–1711. 10.1002/jcb.25827 (2017). 27935095 10.1002/jcb.25827

[60] Kong, J. H. et al. A comparative study on the transplantation of different concentrations of human umbilical mesenchymal cells into diabetic rats. *Int. J. Ophthalmol.* **8** (2), 257–262. 10.3980/j.issn.2222-3959.2015.02.08 (2015). 25938037 10.3980/j.issn.2222-3959.2015.02.08 PMC4413587

[61] Millán-Rivero, J. E. et al. Human wharton’s jelly mesenchymal stem cells protect axotomized rat retinal ganglion cells via secretion of anti-inflammatory and neurotrophic factors. *Sci. Rep.* **8** (1), 16299. 10.1038/s41598-018-34527-z (2018). 30389962 10.1038/s41598-018-34527-z PMC6214908

